# Impact of Rare Sugar D-Allulose on Hardening of Starch Gels during Refrigerated Storage

**DOI:** 10.3390/foods13142183

**Published:** 2024-07-11

**Authors:** Alexandra Obenewaa Kwakye, Kazuhiro Fukada, Toya Ishii, Masahiro Ogawa

**Affiliations:** 1 Faculty of Agriculture, Kagawa University, 2393 Ikenobe, Miki 761-0795, Kagawa, Japan; i742007a@mails.cc.ehime-u.ac.jp (A.O.K.); fukada.kazuhiro@kagawa-u.ac.jp (K.F.); ishii.toya@kagawa-u.ac.jp (T.I.); 2 The United Graduate School of Agricultural Sciences, Ehime University, 3-5-7 Tarumi, Matsuyama 790-8566, Ehime, Japan

**Keywords:** amylose, amylopectin, SEM, FTIR, interaction

## Abstract

The rare sugar D-allulose (Alu), with ca. 10% calories of sucrose (Suc), is a promising alternative sugar that can be used to improve the quality of starch gels in storage. The effects of Alu (compared to Suc) on the hardening and microstructural and molecular order of amylopectin-rich (glutinous rice (GR) and corn amylopectin (CAP)) and amylose-rich (corn (C)) starch gels were investigated. Alu and Suc both suppressed hardening in C gels, while Alu but not Suc was effective in GR and CAP gels. SEM results showed that Alu-containing GR and CAP maintained a relatively large pore size compared to Suc-containing gels. The deconvolution of FTIR spectra revealed that Alu-containing GR and CAP gels had lower ratios of intermolecular hydrogen bonds and higher ratios of loose hydrogen bonds than Suc-containing gels. For amylose-rich C gels, on the other hand, such tendencies were not observed. The influence of Alu on amylopectin-rich gels could be because Alu reduced the ratio of intermolecular hydrogen bonds, which might be involved in amylopectin recrystallization, and increased that of loose hydrogen bonds. The results suggest that Alu is more effective than Suc in inhibiting the hardening of amylopectin-rich starch gels during refrigerated storage.

## 1. Introduction

Carbohydrates, particularly starch and sugars, are common food substances that constitute approximately 70–80% of the total calories consumed globally [1]. During cooking in water, starch undergoes gelatinization to form starch gels; the properties of gelatinized starch gels, such as their textural properties, change during cooling and/or storage because of retrogradation [2,3,4]. Sugars are normally used to reduce undesirable textural changes in starch-based foods that are caused by retrogradation. However, the extent of their effect depends on the type of sugar and other factors, such as the amylose/amylopectin ratio of that particular starch [2,3,5]. Sugars stabilize the crystalline region of starch during gel formation by decreasing the number of water molecules in the hydration co-sphere in a water–starch–sugar system [3]. During cooling, the hydroxyl groups of sugars inhibit retrogradation by binding to the neighboring water molecules as well as to the hydrophilic parts of the starch molecules, leading to limited movements during storage [6]. Among various sugars, sucrose (Suc) is the most commonly used food additive for reducing starch retrogradation, as Suc also possesses additional functions, including sweetening, bulking, texture improvement, and preservation [7]. However, Suc can contribute to metabolic diseases such as obesity and diabetes because of its high calorie count (4 kcal/g) [7].

Rare sugars are “monosaccharides and their derivatives that are rare in nature”, as defined by the International Society of Rare Sugars. Rare sugars are produced from common sugars, polyols, and various building blocks (International Society of Rare Sugars). Examples of rare sugars include L-glucose, D-allulose (Alu), D-allose, D-tagatose, xylitol, etc. Alu, the C-3 position epimer of D-fructose, is a potential substitute for Suc because it has a low calorie count (ca. 10% of the calories of Suc) and has 70% of the sweetness of Suc [8]. Alu has also been found to have physiological benefits, e.g., anti-hyperglycemic and anti-obesity effects [9,10]. Furthermore, Alu is generally recognized as safe (GRAS) for consumption by the US Food and Drugs Authority and was certified by the Japanese Ministry of Consumer Affairs Agency as a food with function in 2022.

In recent years, studies on the application of Alu in food products have been carried out by many research groups. In high-protein foods, for example, it was reported that Alu increased the breaking stress of meringue and custard pudding [11,12], thereby improving the eating quality of the meringue and custard pudding. Other studies also found that compared to Suc, the addition of Alu to a starch-based composite gel improved the gel network and enhanced Maillard browning while suppressing retrogradation [13]. Alu has also been applied to baked goods such as cupcakes. It was reported that compared to Suc, cupcakes containing Alu had higher water-holding capacity, leading to softer cupcakes, but this negatively affected the volume of the cupcakes produced [14]. Another study also found that at similar concentrations (ca. 30–50%), Alu was more effective at inhibiting the retrogradation of wheat starch gels than Suc [15]. However, the possible mechanism of the effects of Alu on suppressing hardening, which occurs as a result of retrogradation, is unclear.

In other starchy foods like bread, although Alu was not utilized by yeast during fermentation, Alu-added white wheat bread was more stable against hardening during storage [16]. Another study on *gyuhi*, a starch-based Japanese sweet prepared using glutinous rice flour and sugar, demonstrated that Alu had the least effect on the gelatinization temperature of glutinous rice flour among various sugars, while delaying retrogradation in storage [17]. These findings in white wheat bread and *gyuhi* indicate the potential use of Alu in starch-rich food systems for suppressing retrogradation. In this context, we previously investigated the effects of Alu on the gelatinization and retrogradation properties of potato, wheat, corn, tapioca, normal rice, and glutinous rice starch sources [18]. The results indicated that Alu had a significant effect on the recrystallization of starch pastes with low amylose/amylopectin ratios, such as normal rice and glutinous rice, but only a minor impact on starch pastes with high amylose/amylopectin ratios.

Based on the above studies, it is expected that Alu would be able to suppress the hardening (an indicator of retrogradation) of amylopectin-rich starch gels but not amylose-rich starch gels, since the coexistence of a large amount of amylose may eliminate the effects of Alu addition. To clarify whether the suppression effect of Alu on starch retrogradation is attributed to interactions between Alu and amylopectin, we used glutinous rice (GR) and corn amylopectin (CAP) starch gels, which contain trace amounts of amylose, as well as amylose-rich corn (C) starch gels as a control in this study. We first evaluated the hardening of the starch gels with/without sugars (Alu or Suc) during refrigerated storage using texture profile analysis and then performed scanning electron microscopy (SEM) and Fourier transform infrared spectroscopy (FTIR) on freeze-dried starch gels to understand the structural changes that possibly occurred in the starch gels with the addition of sugars. 

## 2. Materials and Methods

### 2.1. Materials 

Glutinous rice (GR) flour (Maeharaseifun Co., Ltd., Hyogo, Japan) and corn starch (C) (Maeharaseifun Co., Ltd., Hyogo, Japan) were purchased from a local supermarket. Corn amylopectin (CAP) was obtained from MP Biomedicals LLC (Illkirch, France). D-allulose (Alu) was obtained from Matsutani Industries Ltd., and sucrose (Suc) was obtained from Wako Pure Chemical Industries, Ltd. (Osaka, Japan). Amylose B from corn starch (Nacalai Tesque, Kyoto, Japan) was used for the analysis of total amylose content. All other chemicals used were of analytical grade.

### 2.2. Methods

#### 2.2.1. Moisture Content of the Starch Powders

The moisture contents of GR powder, CAP powder, and C powder were estimated using the oven method. Each sample (1 g) was weighed in a pre-weighed aluminum pan, dried in a convection oven at 105 ± 1 °C until a constant weight was achieved, and then cooled in a desiccator. The moisture content was calculated using the following equation:

(1) $$Moisture\;content\left(\%\right)=\frac{W_{i}-W_{f}}{W_{i}}\times 100$$

where W_i_ is the weight of the starch powder before drying, and W_f_ is the final weight of the starch powder after drying.

#### 2.2.2. Total Starch Content of the Starch Powders

The total amount of starch contained in the starch powder was evaluated using the phenol–sulfuric acid method with slight modifications [19]. An 80% solution of phenol (50 mL) was prepared by topping 40 mL of a concentrated phenol solution with distilled water. Glucose (Glc) standards in different concentrations (0, 0.02, 0.04, 0.06, 0.08, 0.1, 0.12, and 0.14 mg/mL) were prepared from a 1 mg/mL stock solution of Glc. For each starch source, 20 µL of 1 mg/mL starch suspension was pipetted into a glass test tube and 50 µL of 80% phenol was added, followed by 5 mL of concentrated sulfuric acid from an acid dispenser. The final solution was allowed to stand for 10 min, shaken, and incubated in a water bath at 28 ± 1 °C for 15 min. The absorbance of the resultant solution was measured at 490 nm against a reagent blank, and the slope of the standard curve was used to estimate the mass of glucose present. The total starch content was calculated using the equation below.

(2) $$\mathrm{Total}\;\mathrm{starch}\;\mathrm{content}\;\left(\%\right)=\frac{\mathrm{mass}\;\mathrm{of}\;\mathrm{glucose}}{\;\mathrm{mass}\;\mathrm{of}\;\mathrm{starch}\;\mathrm{solution}}\times \;100\;$$

#### 2.2.3. Total Amylose Content of the Starch Powders

The total amylose content of the starch powders was analyzed on defatted starches, as described previously [20]. Standard mixtures of amylose and amylopectin at different ratios from 0 to 100% were prepared, and 20 mg of each and sample starch suspensions were pipetted into a round-bottomed glass test tube. To the standards and samples, 8 mL of 90% dimethyl sulfoxide (DMSO) was added and vortexed for 2 min. The resulting suspensions were heated at 85 °C for 15 min in a water bath with vigorous intermittent mixing, cooled to room temperature, and diluted to 25 mL in a volumetric flask. To determine absorbance, 40 mL of distilled water was added to 1 mL of the diluted starch suspension (and standard solutions), and then 5 mL of iodine solution (0.01 M I_2_/KI) was added. The volume of the solution was adjusted to 50 mL with distilled water, mixed vigorously by hand, and allowed to develop a dark blue color for 15 min. The absorbance at 600 nm was measured against a reagent blank. 

#### 2.2.4. Starch Gel Preparation

**Figure 1:**
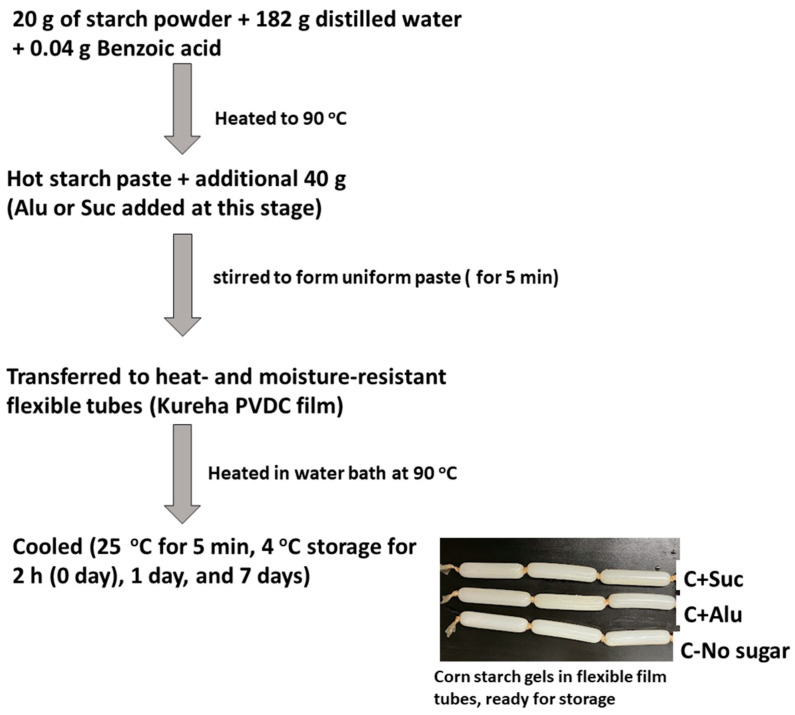
Schematic diagram showing steps used to prepare starch gels with corn starch as an example.

Each starch powder (20 g) was accurately weighed into a flask and mixed with 182 g of distilled water to prepare the starch suspension. Benzoic acid (0.04 g) was added to prevent any possible mold growth during storage. Starch suspensions were gelatinized by heating up to 90 °C in a water bath with intermittent stirring for 5 min. Samples containing Alu or Suc were prepared by adding 18 g of sugar to adjust the starch-to-sugar weight ratio to 10:3. Additional starch (40 g) was added to the hot paste and stirred for 5 min to form a uniform starch paste with a starch concentration of 24.8% (*w*/*w*). The starch pastes were transferred to heat- and moisture-resistant flexible film tubes (Kureha PVDC film) and heated at 90 °C in a water bath for 30 min. They were then cooled in 25 °C tap water for 5 min and stored at 4 °C for 2 h, 1 day, and 7 days to obtain cylindrical gels for analysis. A schematic of the gel preparation steps is illustrated in Figure 1.

##### Texture Analysis

The texture profiles of the prepared starch gels were measured using a Rheoner II creep meter (Yamaden Co., Ltd., Tokyo, Japan). A horizontal cross-section of each cylindrical gel was cut to yield gel samples of 17 mm diameter and 10 mm height. The gel samples were double compressed to 50% of their original height at a compression rate of 1 mm s^−1^ using a 3 cm diameter cylinder probe. For the glutinous rice (GR) and corn amylopectin (CAP) gels, plastic wrap was used to cover the top and bottom of the gel before compressing with a 20 N load cell. The GR and CAP gels were covered to enable ease of measurement because the GR and CAP gels had the tendency to stick to both the stage and probe. The primary parameters needed in this study were hardness and cohesiveness, which were not affected. For the corn starch (C) gels, the texture profile was measured with a 200 N load cell because the texture profile of the C gels after storage at day 1 and day 7 could not be measured due to overload of the 20 N load cell. At least four samples were measured for each starch gel, and hardness (the maximum stress recorded in the first compression cycle; Pa), cohesiveness (the area of work during the second compression divided by the area of work during the first compression; unitless), and gumminess (hardness × cohesiveness; Pa) were calculated automatically using the texture analysis software (model TAS-3305, version 2.4) of the creep meter. 

##### Scanning Electron Microscopy (SEM)

The gel samples, stored in a refrigerator on day 0, day 1, and day 7, were frozen at −79 °C for 2 h in a freezer, followed by freeze drying for 24 h. The micromorphology of the freeze-dried gels was observed using a benchtop scanning electron microscope (JCM-7000 NeoScope™, JEOL, Tokyo, Japan). A horizontal cross-section of each freeze-dried gel sample was cut using a sharp blade. Images were captured in low-vacuum mode with an accelerating voltage of 15 kV and magnification of 400×.

##### Fourier Transform Infrared (FTIR) Spectroscopy

Each freeze-dried gelatinized starch sample was ground to a powder, passed through a 100 mesh sieve, placed in aluminum foil bags containing oxygen absorber and silica gel, and stored in a freezer. Samples (2 mg) and pure potassium bromide tablets (100 mg) were mixed, ground, and pelleted using a manual press to prepare five pellets for each sample. FTIR spectra were obtained using FTIR 670Plus (JASCO Co., Ltd., Tokyo, Japan) equipped with a potassium bromide (KBr) beam splitter and DLATGS detector. The absorption spectra in the range of 4000–400 cm^−1^ at a resolution of 4 cm^−1^ (scanned 64 times) were recorded with the background subtraction for pure KBr. Averaged spectra were obtained from the five pellets, and the following data analysis was performed. The spectra were deconvoluted as a combination of Gaussian components in the range 960–1060 cm^−1^ (3100–3700 cm^−1^) using OriginPro (Version 2024, Origin Lab Corporation, Northampton, MA, USA) for the molecular order (hydrogen bonding pattern) analysis of starch. The spectra were baseline corrected, and the second-derivative spectra were obtained by applying the Savitzky–Golay smoothing method. The ratios of absorbances at 995 cm^−1^/1022 cm^−1^ and 1047 cm^−1^/1022 cm^−1^ were used to estimate the short-range ordered structure of starch [21,22,23].

In the 3100−3700 cm^−1^ region, three bands assigned to different hydrogen-bonded O–H groups were obtained for each sample using deconvolution [23,24]. From the peak positions of the deconvoluted spectrum components, the energies of hydrogen bonds were calculated using the following formula [23,24].

(3) $$E_{H}=\frac{1}{k}\left[\frac{\upsilon_{0}-\upsilon }{\upsilon_{0}}\right]$$

where $\upsilon_{0}$ is the wavenumber of the free hydroxyl group (3650 cm^−1^), υ is the wavenumber of the peak top of each deconvoluted component (cm^−1^), and k is a constant (1/k − 2.625 × 10^2^ kJ). Peak areas of the three bands, on the other hand, were used as a measure of the relative content of the different hydrogen-bonding patterns in the starch samples.

### 2.3. Statistical Analysis

The results of statistical analysis are expressed as mean ± standard deviation with significant differences determined by one-way ANOVA and Tukey’s test (α = 0.05) using IBM SPSS statistics software for Windows, version 28.0 (IBM Corp.; Armonk, NY, USA).

## 3. Results and Discussion 

### 3.1. Composition of the Starch Sources

The compositions of GR, CAP, and C starch sources were investigated to verify the amylose/amylopectin ratio (Table 1). The amount of amylose present was in the order C > GR ≈ CAP, with the corresponding amylose/amylopectin ratio of C > GR > CAP. Starch can be classified based on amylose contents as waxy (0–5% amylose) and normal amylose (20–30% amylose) starch [25,26]; therefore, GR and CAP were classified as waxy and C as normal amylose starches. The contents of amylose and amylopectin in particular play a significant role in the gel-forming properties of the starches. Amylose is a lightly branched linear polymer of α–(1,4)-D–glucopyranosyl units, while amylopectin is a highly branched polymer of α–(1,4)- and α–(1,6)-linked D–glucopyranosyl units. Generally, amylose is a smaller molecule than amylopectin, and the variations in the ratios of the components of starch (amylose and amylopectin) affect gelatinization and recrystallization upon heating and cooling, respectively [27]. Previous studies demonstrated that higher contents of amylose in starch gels were responsible for the gel-forming ability of starch due to the ability of amylose to leach out of starch granules and form networks during gelatinization [28]. Generally, amylose also recrystallizes faster than amylopectin [29], therefore, the different components of GR, CAP, and C impact their characteristics. 

### 3.2. Effects of Alu on the Textural Properties of the Starch Gels

**Figure 2:**
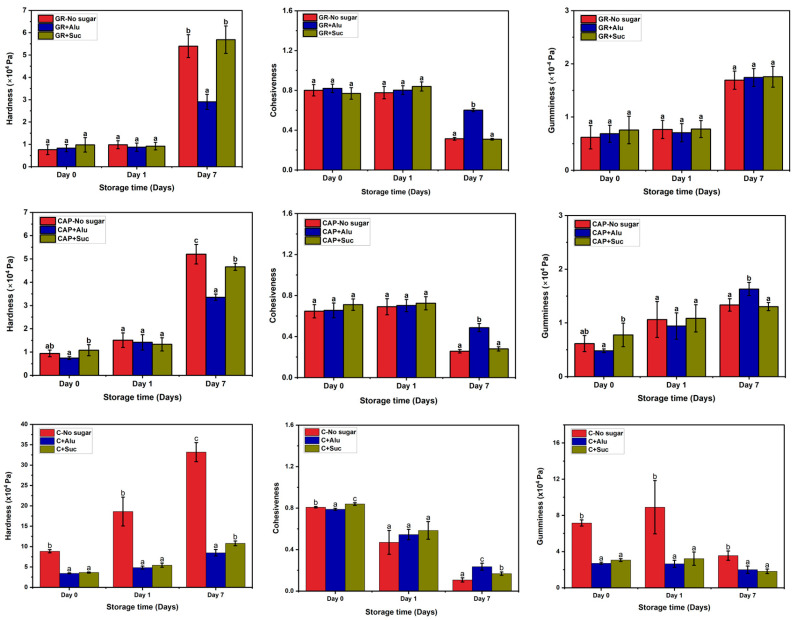
Hardness, cohesiveness, and gumminess of the starch gels and their changes during storage at 4 °C. Different letters indicate significant differences (*p* < 0.05) between different samples in the same period. GR and CAP gels were measured using a 20 N load cell, while C gels were measured using a 200 N load cell.

Figure 2 shows the results of texture analysis of the starch gels. The hardness of all the gel samples increased during the refrigerated storage period. On day 0 and day 1, the hardness of GR and CAP gels containing Alu or Suc was not significantly different from that with no sugar. However, both sugars showed an inhibitory effect on the hardening of C gels during storage. In all the starch gels, the most notable variation in hardness was observed on day 7. Alu had an inhibitory effect on hardening in GR and CAP, but Suc had almost no effect. In the C gels, the inhibitory effect of Alu was slightly higher than that of Suc. There were no significant differences between the cohesiveness of GR and CAP gels containing Alu and Suc on day 0 and day 1 of storage. For the C gels with/without sugar, there was no significant difference in the cohesiveness on day 1. On day 7, however, the cohesiveness of all the starch gels decreased during storage, and the cohesiveness of starch gels containing Alu was almost two times higher than that if no-sugar or Suc-containing gels. These results suggest that Alu was effective in preventing the starch gels from losing cohesiveness in longer 7 day storage, while Suc had almost no effect. The gumminess of GR and CAP gels increased during storage, with no significant difference in the gels with/without sugar except for CAP containing Alu on day 7. The gumminess of C gels was generally reduced by the addition of Alu or Suc.

The lower hardness of the GR and CAP gels containing Alu observed after 7 days of storage indicated the anti-retrogradation effect of Alu. Other authors reported that the addition of Alu and Suc suppressed the hardening of GR gel during refrigerated storage and that Alu had a stronger suppression effect than Suc [17,30]. Additionally, previous studies have suggested that Alu has a higher water-holding capacity than Suc when used in surimi gels and cupcakes [14,31]. Furthermore, the amount of unbound water in *gyuhi* containing Alu was found to be lower than that in *gyuhi* containing Suc after storage, indicating better retrogradation suppression [17]. Starch retrogradation is a phenomenon that occurs during the process of the realignment of disordered amylose and amylopectin chains in gelatinized starch, leading to a dense and hard gel [2,3,4,5,32,33]. During cooling and/or storage, hydrogen bonding interactions between Alu or Suc, water, amylose, and amylopectin components of the starches possibly influenced the effects on the hardening of the starch gels. The results in Figure 2 suggest that Alu had a stronger ability to prevent the realignment of both amylose and amylopectin chains compared to Suc.

Cohesiveness is the ability of a sample to retain its original shape after initial compression and a measure of the stickiness of the internal structure [34,35]. If bonds in the internal structure are broken during the initial compression, there will be a smaller chance of recovery during the next compression [35]. Alu had a greater ability to prevent the internal bonds of all the starch gels from breaking compared to that of Suc and no sugar, especially after 7 days of storage. GR and CAP are waxy starches, while C is a normal amylose starch. Alu possibly interferes with both amylose and amylopectin recrystallization during long-term storage, resulting in softer gels. Suc seems to be effective in suppressing hardness but to a lower extent than Alu. Alu appears to be effective in suppressing not only amylose recrystallization (short-term) but also amylopectin recrystallization (long-term), resulting in a higher anti-retrogradation ability.

### 3.3. Microstructure of the Starch Gels during Storage

To understand the possible causes of changes in the texture of the refrigerated starch gels, the microstructures of the freeze-dried starch gels were observed using SEM. It is to be noted that, even though the starch gels were frozen at −79 °C before freeze drying, retrogradation was unlikely to occur. This is because starch chains have limited mobility at the extremely low freezing temperature. Therefore, only the “free water” available in the starch gels froze and formed ice crystals [36]. 

**Figure 3:**
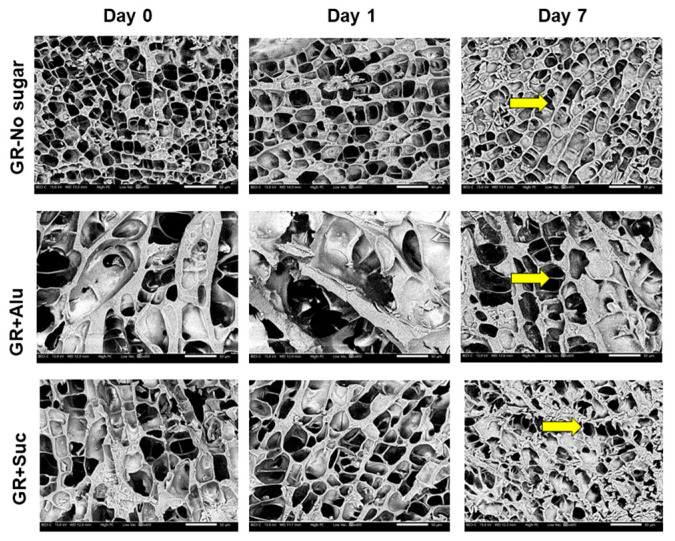

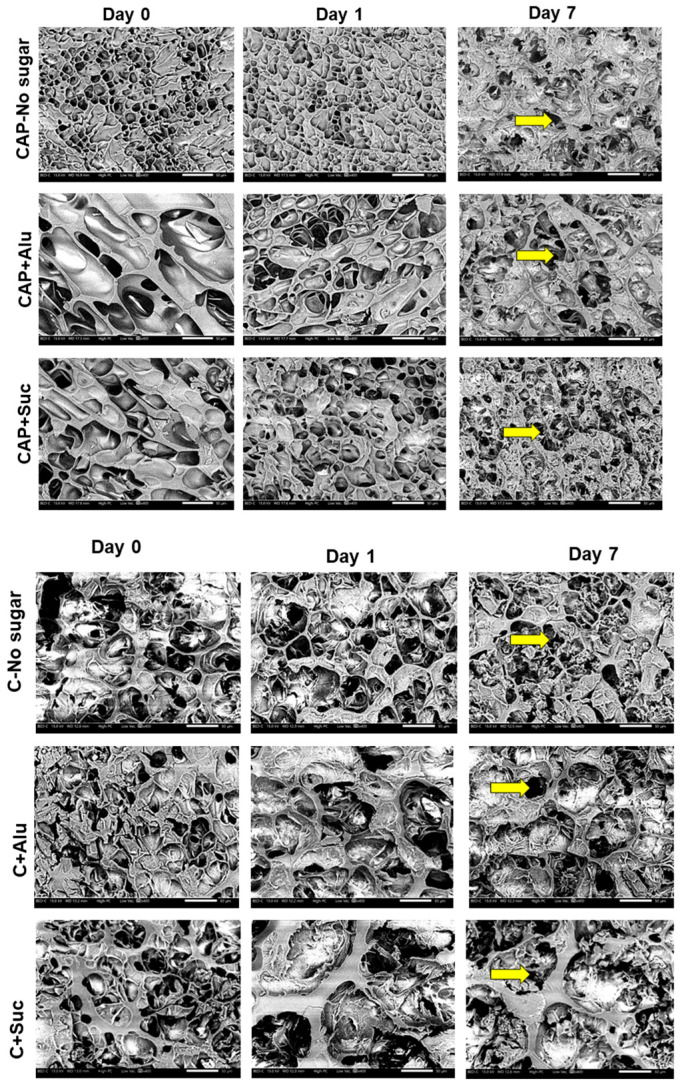
Scanning electron micrographs of freeze-dried starch gels stored at 4 °C. Yellow arrows represent the pores present in the respective starch gels. Magnification: 400×; scale bar: 50 μm.

Figure 3 shows the SEM micrographs of the starch gels after refrigerated storage. On day 0, GR containing Alu had the largest pores, followed by GR containing Suc and no sugar. A similar pattern was observed for CAP. For C on day 0, however, the pore sizes were almost the same with and without sugar. After one day of storage, GR containing Alu showed particularly larger pores than CAP and C with Alu. The pore sizes of GR, CAP, and C containing Suc were reduced on day 1. On day 7, GR and CAP containing Alu maintained a relatively large pore size compared with that of GR and CAP with no sugar and Suc. In contrast, for C on day 7, the pore sizes were still almost the same with and without sugar. 

Previous studies reported that starch gels containing higher amounts of amylose form a dense microstructure, while those with low amylose form a loose microstructure [28]. These observations were similar to those observed in GR, CAP, and C. The microstructures of GR and CAP containing Alu were different from those with no sugar and Suc. GR and CAP containing Alu may have retained more “freezable water” [36] than GR and CAP containing Suc. Unlike GR and CAP, C containing Alu was similar to that with no sugar and that with Suc. Differences could not be observed in the C gels, possibly due to the random and fast recrystallization of amylose characterized by the higher load cell (200 N) required for texture analysis. Previous studies suggested that high concentrations of Suc (up to 20%) stabilized the microstructure of rice starch gels with ca. 31% amylose content (during freeze–thaw treatment) by maintaining the matrix around the pores [37]. On day 7, in particular, Alu possibly reduced the reassociation of the amylopectin starch chains in GR and CAP, but not in C. The observations of GR and CAP containing Alu indicate that Alu possibly has a better microstructure-stabilizing effect in amylopectin-rich starch gels than Suc. The results from the SEM micrographs correlate well with the hardening-suppression effect of Alu observed in the GR and CAP starch gels during refrigerated storage, especially on day 7. 

### 3.4. FTIR of Freeze-Dried Starch Gels (Starch Region)

**Figure 4:**
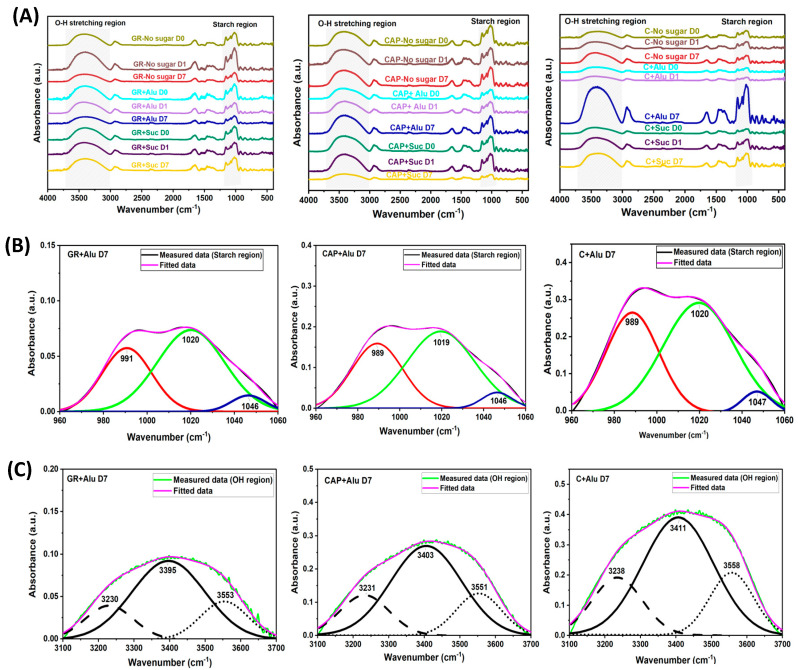
The FTIR spectra of freeze-dried GR, CAP, and C gel powders. (**A**) Absorbance spectra at 4000–400 (cm^−1^). (**B**) The deconvoluted FTIR spectra of the starch region of GR, CAP, and C containing Alu on D7. (**C**) The deconvoluted FTIR spectra of the O–H stretching region of GR, CAP, and C containing Alu on D7. The freeze-dried starch gels were ground to powder and passed through a 100 mesh sieve before measurements. The three peaks were consistent in all the other samples for the O–H stretching region and starch region.

FTIR was conducted to understand the changes in the molecular order and hydrogen bonding in the starch gels during refrigerated storage. The FTIR spectra of the freeze-dried starch gels are shown in Figure 4A. In all the starch samples, sharp IR bands around 960–1060 cm^−1^ assigned to the starch region and broad IR bands around 3100–3700 cm^−1^ assigned to the O–H stretching region were detected [23,38,39]. It is known that these bands are composed of overlapping components; hence, the second derivatives of the FTIR spectra were used to identify the characteristic peaks of the components. Three peaks in the starch region and the O–H stretching region were obtained from Gaussian analyses, respectively. Figure 4B,C shows examples of spectra obtained from starch gels containing Alu on day 7.

**Figure 5:**
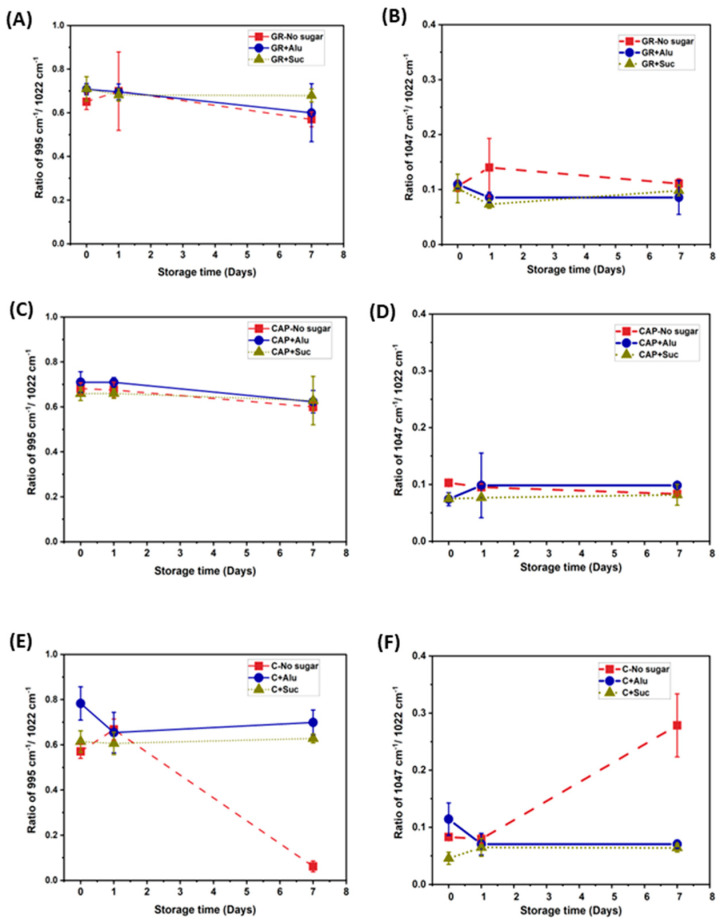
Changes in the short-range molecular order of the starch gels during the storage period. (**A**,**C**,**E**)—Absorbance ratio at 995 cm^−1^/1022 cm^−1^. (**B**,**D**,**F**)—Absorbance ratios at 1047 cm^−1^/1022 cm^−1^.

The IR absorption bands at 995 and 1047 cm^−1^ have been reported to be related to the crystalline regions of starch [23,38,40], and the absorption band at 1022 cm^−1^ can be related to the amorphous region [23,40,41]. The ratio of absorbance at 995 cm^−1^/1022 cm^−1^ represents the degree of double helices (DD), and that at 1047 cm^−1^/ 1022 cm^−1^ represents the degree of order (DO) [21,22]. Hence, the ratios of absorbance at 995 cm^−1^/1022 cm^−1^ and 1047 cm^−1^/ 1022 cm^−1^ are good indicators of short-range molecular structural changes in the starch gels. To determine the changes in molecular order of the starch gels during storage, these absorbance ratios were calculated and are shown in Figure 5. For GR and CAP, all the samples containing Alu, Suc, or no sugar showed no significant differences in either DD or DO (Figure 5A–D). The only notable change was observed for C on day 7 in Figure 5E, where the DD of the no sugar gel decreased during storage while that with Alu or Suc did not change on day 7. On the other hand, the DO for C gels with no sugar increased during storage, while that for C with Alu or Suc did not change (Figure 5F). The observations of the DO for the C gels were similar to those in other studies, where DO increased with storage time for starch gels stored at refrigerated temperatures for 7 days [23,42]. In the amylose-rich C gels, the recrystallized amylose served as seed nuclei for amylopectin recrystallization [43]. These results suggest that the DD and DO of C gels without sugars changed drastically after storage, while Alu and Suc prevented the starch chain structure from changing during storage, implying that sugar addition suppressed the recrystallization of C gels. These results may suggest that under freeze-dried conditions, Alu or Suc can stabilize molecular order of amylose-rich starch gels for longer storage. 

### 3.5. FTIR of Freeze-Dried Starch Gels (O–H Stretching Region)

Starch has three types of O–H groups involved in hydrogen bonding [44]. The infrared absorption of O–H stretching is reported to be between 3000–3700 cm^−1^, which is typical for carbohydrates, cellulose, and starch [23,24,45]. Brubach and others analyzed the FTIR spectra of the O–H stretching region and reported that there were three dominant components [40,46]. The component with the lowest wavenumber (3295 cm^−1^) was assigned as network water because it was similar to that of ice. The highest wavenumber (3640 cm^−1^) was assigned as multimer water, representing loosely connected water and free O–H groups. The middle wavenumber (3460 cm^−1^) was assigned to intermediate water between these two states. Other authors referred to the bonds in these regions as intermolecular H-bonds (3200–3300 cm^−1^), intramolecular bonds (~3400–3500 cm^−1^), and loosely connected H-bonds (above 3500 cm^−1^) [23,24]. The changes in the hydrogen bonds of starch also influence starch retrogradation [23]. Since starch gels are formed by amylose and/or amylopectin chains bonding with water via hydrogen bonds to form a network structure, analysis of the O-H stretching region can be used to interpret the properties of the network structure and changes that occur during storage. 

**Figure 6:**
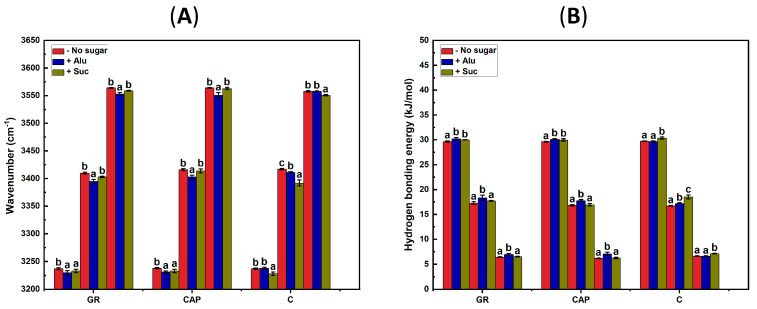
Evolution of hydrogen bonds in the O-H stretching region. (**A**) Peak center positions in the GR, CAP, and C gels on storage day 7. (**B**) Hydrogen bonding energy. Intermolecular H-bonds (Inter), intramolecular H-bonds (Intra), and loose H-bonds (Loose). Different letters indicate significant differences (*p* < 0.05) between different samples around the same peak.

A peak shift to a lower wavenumber in these regions indicates a stronger hydrogen bond (i.e., higher bonding energy) [24,45]. The fitted peak centers and estimated hydrogen bonding energies for all the starch gels on day 7 of storage are depicted in Figure 6. On day 0 and day 1, no significant trend in shifting of the fitted peak centers and corresponding bonding energies with Alu or Suc addition was confirmed (Table S1). On day 7, however, Alu shifted the peak centers of all three types of hydrogen bonds in GR and CAP to a lower wavenumber (Figure 6A), resulting in increased bonding energies for intermolecular, intramolecular, and loose hydrogen bonds (Figure 6B). In C containing Alu, the peak centers of only the intramolecular bonds shifted to a lower wavenumber. On the other hand, the presence of Suc caused the peak centers of all types of bonds to shift to a lower wavenumber in C, resulting in increased bonding energies. 

The tendency observed in Figure 6, particularly for the waxy samples (i.e., GR and CAP), corresponded well to the gel hardness results discussed earlier (Figure 2). This implies that Alu exhibited notable effects on both the texture and hydrogen bonds in the refrigerated waxy gels, but Suc did not. These results suggest that the ability of Alu to maintain the texture of the refrigerated waxy gels could be attributed to a relative dominance of these types of hydrogen bonds in each sample. The relative contents of the intermolecular, intramolecular, and loose hydrogen bonds of the starch gels were therefore calculated from day 0 to day 7 (Figure S1). However, the ratio of these hydrogen bonds in each sample did not exhibit clear time-dependent trends, unlike the changes in the gel hardness during the storage. This result was probably because of the complex interactions between starch, sugar, and water molecules that occur during storage [2]. The presence of amylose, which has different retrogradation kinetics from amylopectin, could have also affected the results obtained, even though amylose was present in trace amounts in GR and CAP.

**Figure 7:**
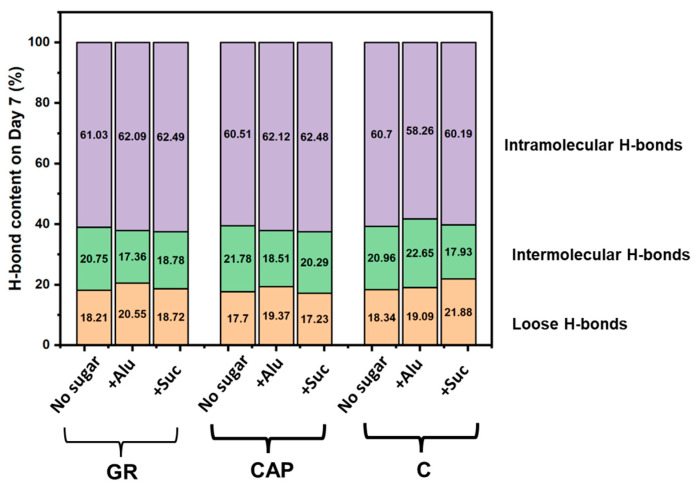
Hydrogen bond contents (%) of freeze-dried GR, CAP, and C gels on storage day 7.

To obtain an insight into the interactions of gels stored on day 7, the hydrogen bond contents of the day 7 samples are depicted in Figure 7. This is because the retrogradation of both amylose and amylopectin possibly progressed significantly on day 7. Notable differences in the ratios of H-bonds were observed for GR and CAP containing Alu. GR and CAP containing Alu had lower intermolecular H-bond (approx. 17~18%) and higher loose H-bond (approx. 20%) ratios than the GR and CAP with no sugar and Suc. Amylopectin recrystallizes at a slower rate than amylose [29] and possibly recrystallized the most on day 7 of storage. The transition from intermolecular to loose H-bonds by the addition of Alu suggests that Alu possibly interfered with interactions between amylopectin chains during storage. For amylose-rich C gels, on the other hand, such tendencies were not observed, and the addition of Alu seemed to instead increase the relative dominance of intermolecular H-bonds in comparison with the no-sugar and Suc samples.

These analyses indicate that, compared with the no-sugar samples, Alu addition induced a transition from intermolecular H-bond to loose H-bond (Figure 6) in the amylopectin-rich GR and CAP gels on day 7 of storage, but Suc addition did not cause such phenomena. The ability of Alu to enhance the H-bond amounts and reduce the relative content of the intermolecular H-bonds in the GR and CAP gels may have led to higher hardening suppression observed on day 7 compared to Suc. Suc molecules in aqueous solution contain an intramolecular H-bond between the glucose and fructose moieties [47]. This may have contributed to the weaker effect of Suc observed because it may be less interactive with starch and water molecules via H-bonds than Alu at an equal weight-based concentration. In addition, our previous study reported that Alu suppressed the retrogradation of 2% GR starch pastes more effectively than not only Suc but also other monosaccharides [18]. The molecular orientation of Alu might be favorable for interactions between Alu and amylopectin molecules, possibly resulting in the different characteristics of H-bonds in starch gel systems. On the contrary, the addition of Alu to C gel increased the amount of intermolecular H-bonds compared to C gel with no sugar or Suc. These results suggest that Alu’s retrogradation suppression mechanism for amylopectin-rich starch gels is different from that for amylose-rich starch gels. 

## 4. Conclusions

The present research showed that Alu significantly suppressed the hardening of amylopectin-rich starch gels during refrigerated storage more than Suc. The influence of Alu on amylopectin-rich gels could be because Alu reduced the ratio of intermolecular hydrogen bonds, which might be involved in amylopectin recrystallization, and increased that of loose hydrogen bonds. In addition to its effects on the amylopectin-rich gels, Alu also exhibited a stronger suppression effect on the gel hardening of amylose-rich gels than Suc, although the molecular mechanisms seem to be different from those observed in amylopectin-rich gels. Suc is the most widely used sugar in the food industry because it has many food applications that have not been replicated by any other sugar [7]. Our findings demonstrate that Alu is particularly capable of suppressing the hardening of amylopectin-rich starch gels and may also be more effective than Suc, suggesting the potential of Alu as a food ingredient for various starch-based foods. Consumers will possibly also benefit from the physiological benefits of Alu in starch-based foods containing Alu.

## Figures and Tables

**Table 1:** Major components of the starches.

Starch Source	Moisture (%)	Total Starch Content (%)	Total Amylose Content (%)	Amylose/Amylopectin Ratio
Glutinous Rice (GR)	11.07 ± 0.04 ^b^	82.27 ± 3.21 ^a^	2.13 ± 0.21 ^a^	1/80
Corn Amylopectin (CAP)	9.86 ± 0.10 ^a^	82.27 ± 3.21 ^a^	0.28 ± 0.01 ^a^	1/290
Corn (C)	11.05 ± 0.10 ^b^	81.65 ± 4.92 ^a^	27.04 ± 1.53 ^b^	1/2

Level of significance (α = 0.05). Values with the same letters in the same column are not significantly different. Amylose/amylopectin ratio is the ratio of total amylose content divided by amylopectin content, which was calculated by subtracting total amylose content from total starch content.

## Data Availability

The original contributions presented in the study are included in the article; further enquiries can be directed to the corresponding author.

## Supplementary Materials

The following supporting information can be downloaded at: https://www.mdpi.com/article/10.3390/foods13142183/s1, Figure S1: Hydrogen bond contents (%) of freeze-dried GR, CAP, and C gels on storage days 0, 1, and 7; Table S1: Hydrogen bond energies in the freeze-dried starch gel powders.
