# Supplementary material for: Impact of Rare Sugar D-Allulose on Hardening of Starch Gels during Refrigerated Storage
Source: Foods. 2024 Jul 11;13(14):2183. doi: 10.3390/foods13142183 (PMC11275381; doi:10.3390/foods13142183)
Supplement: Supplementary file 1 [file foods-13-02183-s001.zip › foods-3080529-supplementary.pdf]

Supplementary materials

Impact of rare sugar D-allulose on hardening of starch gels during refrigerated storage

Alexandra Obenewaa Kwakye[1,2], Kazuhiro Fukada[1], Toya Ishii[1], Masahiro Ogawa[1*]

[1]Faculty of Agriculture, Kagawa University, 2393 Ikenobe, Miki, Kagawa, 761-0795, Japan
[2]The United Graduate School of Agricultural Sciences, Ehime University, 3-5-7 Tarumi, Matsuyama, Ehime, 790-8566, Japan

*Author to whom correspondence should be addressed. e-mail: ogawa.masahiro@kagawa-u.ac.jp, Fax: +81-87-891-3021

Table S1: Hydrogen bond energies in the freeze-dried starch gel powders

Sample	Intermolecular H-bond		Intramolecular H-bond		Loose H-bond	
	Fitted peak center (cm^{-1})	E_H (kJ/mol)	Fitted peak center (cm^{-1})	E_H (kJ/mol)	Fitted peak center (cm^{-1})	E_H (kJ/mol)
**GR-No sugar D0**	3238	29.625	3411	17.177	3559	6.566
**GR+Alu D0**	3226	30.503	3390	18.690	3547	7.398
**GR+Suc D0**	3232	30.037	3402	17.848	3552	7.044
**GR-No sugar D1**	3234	29.952	3402	17.831	3556	6.782
**GR+Alu D1**	3237	29.693	3413	17.056	3556	6.748
**GR+Suc D1**	3234	29.938	3408	17.379	3559	6.574
**GR-No sugar D7**	3237	29.688	3410	17.286	3561	6.423
**GR+Alu D7**	3230	30.211	3395	18.347	3553	7.012
**GR+Suc D7**	3233	30.024	3403	17.764	3559	6.541
**CAP-No sugar D0**	3237	29.725	3411	17.164	3558	6.595
**CAP+Alu D0**	3237	29.667	3418	16.703	3558	6.597
**CAP+Suc D0**	3237	29.720	3413	17.039	3557	6.685
**CAP-No sugar D1**	3236	29.740	3415	16.877	3565	6.115
**CAP+Alu D1**	3230	30.195	3405	17.645	3549	7.263
**CAP+Suc D1**	3232	30.050	3407	17.478	3554	6.911
**CAP-No sugar D7**	3238	29.631	3416	16.847	3564	6.190
**CAP+Alu D7**	3231	30.121	3403	17.771	3551	7.098
**CAP+Suc D7**	3233	29.985	3414	16.937	3563	6.273
**C-No sugar D0**	3235	29.859	3404	17.717	3559	6.553
**C+Alu D0**	3230	30.174	3403	17.784	3556	6.790
**C+Suc D0**	3243	29.298	3427	16.004	3568	5.911
**C-No sugar D1**	3236	29.765	3416	16.814	3566	6.029
**C+Alu D1**	3249	28.815	3431	15.727	3565	6.087
**C+Suc D1**	3241	29.394	3420	16.509	3564	6.211
**C-No sugar D7**	3237	29.736	3417	16.732	3558	6.628
**C+Alu D7**	3238	29.637	3411	17.213	3558	6.621
**C+Suc D7**	3228	30.373	3392	18.537	3551	7.138

*E_H is the hydrogen bonding energy

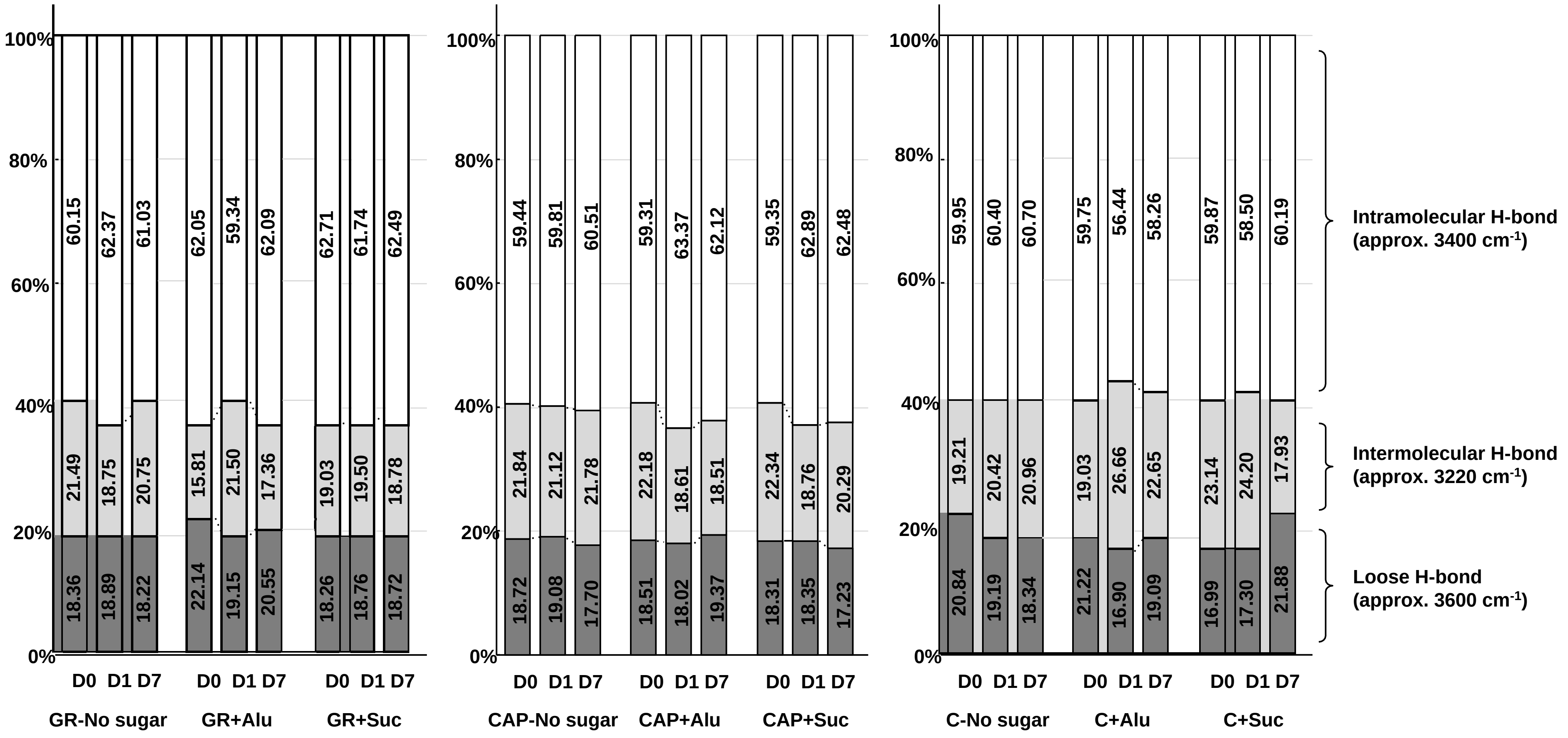

Figure S1: Hydrogen bond contents (%) of freeze-dried GR, CAP, and C gels on storage days 0, 1, and 7
